# The complete mitochondrial genome of the sea spider *Achelia bituberculata *(Pycnogonida, Ammotheidae): arthropod ground pattern of gene arrangement

**DOI:** 10.1186/1471-2164-8-343

**Published:** 2007-10-01

**Authors:** Shin-Ju Park, Yong-Seok Lee, Ui Wook Hwang

**Affiliations:** 1 Department of Biology, Teachers College, Kyungpook National University, Daegu 702-701, Korea

## Abstract

**Background:**

The phylogenetic position of pycnogonids is a long-standing and controversial issue in arthropod phylogeny. This controversy has recently been rekindled by differences in the conclusions based on neuroanatomical data concerning the chelifore and the patterns of *Hox *expression. The mitochondrial genome of a sea spider, *Nymphon gracile *(Pycnogonida, Nymphonidae), was recently reported in an attempt to address this issue. However, *N. gracile *appears to be a long-branch taxon on the phylogenetic tree and exhibits a number of peculiar features, such as 10 tRNA translocations and even an inversion of several protein-coding genes. Sequences of other pycnogonid mitochondrial genomes are needed if the position of pycnogonids is to be elucidated on this basis.

**Results:**

The complete mitochondrial genome (15,474 bp) of a sea spider (*Achelia bituberculata*) belonging to the family Ammotheidae, which combines a number of anatomical features considered plesiomorphic with respect to other pycnogonids, was sequenced and characterized. The genome organization shows the features typical of most metazoan animal genomes (37 tightly-packed genes). The overall gene arrangement is completely identical to the arthropod ground pattern, with one exception: the position of the *trnQ *gene between the *rrnS *gene and the control region. Maximum likelihood and Bayesian inference trees inferred from the amino acid sequences of mitochondrial protein-coding genes consistently indicate that the pycnogonids (*A. bituberculata *and *N. gracile*) may be closely related to the clade of Acari and Araneae.

**Conclusion:**

The complete mitochondrial genome sequence of *A. bituberculata *(Family Ammotheidae) and the previously-reported partial sequence of *Endeis spinosa *show the gene arrangement patterns typical of arthropods (*Limulus*-like), but they differ markedly from that of *N. gracile*. Phylogenetic analyses based on mitochondrial protein-coding genes showed that Pycnogonida may be authentic arachnids (= aquatic arachnids) within Chelicerata *sensu lato*, as indicated by the name 'sea spider,' and suggest that the Cormogonida theory – that the pycnogonids are a sister group of all other arthropods – should be rejected. However, in view of the relatively weak node confidence, strand-biased nucleotide composition and long-branch attraction artifact, further more intensive studies seem necessary to resolve the exact position of the pycnogonids.

## Background

Pycnogonids, or sea spiders, comprising about 1163 extant species [1], are a special group of exclusively marine arthropods that are distributed from the intertidal zone to the abyssal depths in all the seas around the world. The pycogonids are traditionally classified as a sister group or even an ingroup taxon of euchelicerates, which comprises the arachnids, xiphosurans and extinct eurypterids [2,3]. However, the phylogenetic position of pycnogonids has been vigorously debated during the last two centuries, as summarized in a recent review of pycnogonid affinities by Dunlop and Arango [4]. According to this review, there have been a number of controversial hypotheses related to this issue. The pycnogonids have been regarded as either degraded crustaceans [5,6], a transitional form between crustaceans and arachnids [7-9], an isolated group unrelated to other arthropods [10-12], a sister group of aquatic arachnids or mites within Chelicerata [13-15], a sister group of euchelicerates [3,4,16,17], or a sister group of all other extant euarthropods (encompassing chelicerates, myriapods, crustaceans and hexapods) [18-20]. Because of rapid methodological improvements in phylogenetic systematics and related fields such as developmental biology, molecular biology and computational biology (bioinformatics), recent phylogenies have continuously attempted to resolve this issue and have eliminated many of the possible hypotheses, leaving only the two most plausible to be examined: namely that pycnogonids are either a sister group of euchelicerates or a sister group of euarthropods.

Pycnogonids have continually been recovered as a sister group of euchelicerates not only by a convincing autapomorphy – 'chelicerae/chelifore'- between the pycnogonids and euchelicerates, but also by a number of cladistic and phylogenetic studies based on molecular data (DNA or amino acid sequences), combined morphological and molecular data, *Hox *expression data and immunohistochemical data [4,21-32].

On the other hand, the hypothesis that the pycnogonids are a sister group of euarthropods (all extant arthropods except for pycnogonids) was initially formally proposed by Zrzavy et al. on the basis of all the available morphological and gene sequence data [18]. They named the clade of euchelicerates and mandibulates (excluding Pycnogonida) "Cormogonida;" they share the putative autapomorphic characteristic of a gonopore on the trunk. Pycnogonids have multiple gonopores on the bases of their legs. Although some authors have recovered this relationship [14,19,20,33,34], the presence of gonopores on the trunk in Cormogonida has been doubted as a plesiomorphic character [4].

Interestingly, a detailed neuroanatomical study of the *Anoplodactylus *sp. protonymphon lava [20] suggested that the chelifore of euchelicerates is not homologous to the chelicerae of pycnogonids, but rather that it is homologous to the protocerebral appendages ("great appendages") proposed for ancestral arthropods [35,36]. These authors suggested that the pycnogonids are a sister group of all the euarthropods. However, more recent studies of *Hox *expression patterns in *Endeis spinosa *[29,30] refuted the result of the neuroanatomical study [20]. The *Hox *expression data suggested that all the anterior-most appendages of all extant arthropods (chelifore of pycnogonids, chelicerae of euchelicerates and the first antennae of mandibulates) are homologous and deutocerebral, indicating that protocerebral appendages have been lost in all extant arthropods.

In recent years, comparison of complete mitochondrial genomes has become a very powerful tool for reconstructing arthropod phylogeny [15,37-41]. Mitochondrial genomes contain a variety of useful phylogenetic information, such as gene orders and orientations, alternative start codons of protein-coding genes, transfer RNA and ribosomal RNA secondary structures, genetic code variations, and features of the control region for genome replication and transcription [42]. Typical metazoan mitochondrial genomes are circular, 14–16 kb in size, and encode 13 proteins, large and small subunit ribosomal RNAs (*rrnL *and *rrnS*) and 22 tRNAs [38,43]. The 13 polypeptides are involved in ATP synthesis coupled to electron transfer during O_2 _consumption [ATP synthetase subunits (*atp6 *and *atp8*), cytochrome C oxidase subunits (*cox1*-*cox3*), apocytochrome b (*cob*) and NADH dehydrogenase subunits (*nad1*-*nad6 *and *nad4L*)].

Approximately 1014 complete mitochondrial genome sequences have been determined to date from metazoa including 243 protostomes (126 arthropods, 21 nematodes, 4 annelids, 33 mollusks, 16 platyhelminthes, 3 brachiopods, 1 echiuran, 1 bryozoan, 1 acanthocephalan and 2 chaetognaths, 24 cnidarian, 1 onychophoran, 4 placozoan, 5 poriferan, 1 priapulidan) and 771 deuterostomes (752 chordates, 16 echinoderms and 2 hemichordates, 1 xenoturbella) (GenBank status on June, 2007). Of the metazoan mitochondrial genomes sequenced, 126 were from arthropods (65 hexapods, 34 crustaceans, 4 myriapods and 23 chelicerates). In pycnogonids, a partial mitochondrial genome for *E. spinosa *(family Endeidae) was reported by Hassanin [15] and a complete mitochondrial genome sequence for *N. gracile *(family Nymphonidae) by Podsiadlowski and Braband [44]. According to these recent reports, phylogenetic analyses of protein-coding genes show that pycnogonids may be associated with Acari (ticks and mites), although the authors acknowledged that the result may be due to a long-branch attraction artifact and higher A+T content.

The *N. gracile *mitochondrial genome has a peculiar gene order with extensive inversion of protein-coding genes and translocations of 10 tRNA genes, which are not typically found in arthropods [44]. These peculiar characteristics are probably restricted to the *N. gracile *lineage, since the partial *E. spinosa *mitochondrial genome possesses the arthropod ground pattern of mitochondrial gene arrangements. Furthermore, the *N. gracile *branch on the phylogenetic trees presented by Podsiadlowski and Braband [44] was very long, as was also the case for Acari. This may have given rise to an artifactual relationship between Acari and Pycnogonida, which suggests that *N. gracile *may not be an appropriate representative of the pycnogonids. Thus, additional pycnogonid mitochondrial genomes must be sequenced if such information is to be used to address the problem of the phylogenetic position of pycnogonids [45,46].

In this study, we present a complete new pycnogonid mitochondrial genome from a sea spider, *Achelia bituberculata*, belonging to the family Ammotheidae, which combines a number of anatomical features considered plesiomorphic with respect to other pycnogonids. The genome is characterized and compared to those of other arthropods, including two pycnogonids, *N. gracile *and *E. spinosa*. We attempt to use the data obtained in this study to address the long-standing and hotly-debated issue of the phylogenetic position of pycnogonids.

## Results and discussion

### Genome organization

**Figure 1:**
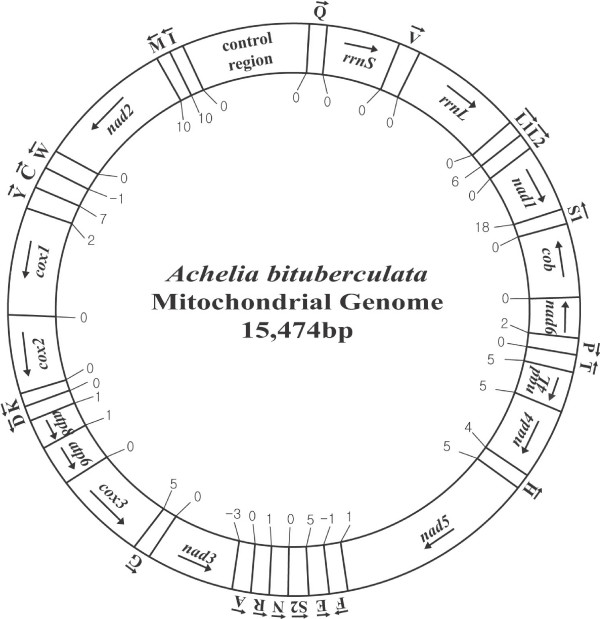
**A circular map of the complete mitochondrial genome of the sea spider *Achelia bituberculata *(Family Ammotheidae)**. Protein and rRNA genes are abbreviated as follows: *atp6 *and *atp8 *(genes for ATPase subunits 6 and 8), *cox1*-*cox3 *(genes for cytochrome C oxidase subunits I-III), *cob *(gene for apocytochrome b), *nad1*-*nad6 *and *nad4L *(genes for NADH dehydrogenase subunits 1–6 and 4L), and *rrnS *and *rrnL *(genes for 12S and 16S rRNAs). All the 22 tRNA genes are located among protein- and/or tRNA-coding genes. The tRNA genes are named using single-letter amino acid abbreviations, with the exception of those coding for leucine and serine, which are named *L*1 for the tRNA^Leu(UUR) ^(anticodon TAA) gene, *L*2 for the tRNA^Leu(CUN) ^(anticodon TAG) gene, *S*1 for the tRNA^Ser(UCN) ^(anticodon TGA) gene and *S*2 for the tRNA^Ser(AGN) ^(anticodon GCT) gene. The arrows indicate the orientations of the gene components.

**Figure 2:**
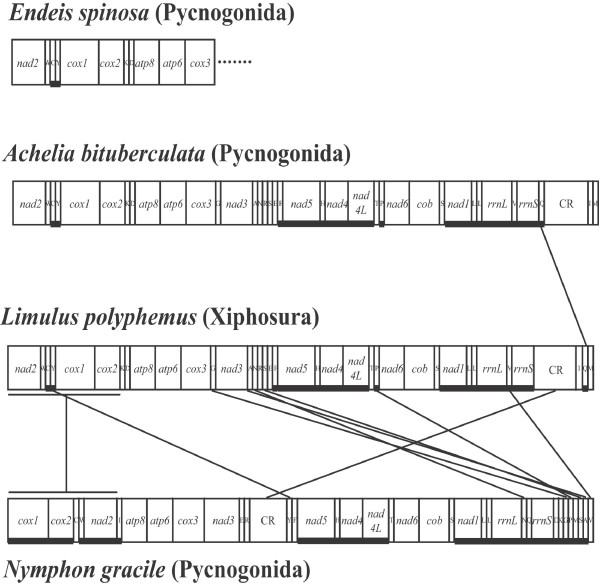
**Comparison of the mitochondrial gene arrangements of three sea spiders, *Achelia bituberculata*, *Endeis spinosa *and *Nymphon gracile*, and a horseshoe crab, *Limulus polyphemus*, which is considered to be an arthropod ground pattern**. The mitochondrial gene arrangements in *A. bituberculata *(complete) and *E. spinosa *(partial) are the same as the arthropod ground pattern shown in *L. ployphemus*, except for the unexpected placement of *trnQ *(Q) in *A. bituberculata*. In *N. gracile*, however, the gene arrangement differs markedly from the other two pycnogonids. The two *trnL1 *and *trnL2 *genes are marked by *L*1 and *L*2, respectively. *L*1 and *L*2 in *A. bituberculata *and *N. gracile *have the orientation and position generally shown in the chelicerates and myriapods among the arthropods. Refer to the Fig. 1 legend for abbreviations of components.

The mitochondrial genome of *A. bituberculata *is 15,474 bp in length, which is similar to those of arthropod mitochondrial genomes (Table 1). It includes 13 protein-coding genes (*cox1*-*cox3*, *nad1*-*nad6*, *nad4L*, *atp6*, *atp8 *and *cob*), 22 tRNA genes (*trnA*, *trnR *etc.), two ribosomal RNA genes (*rrnL *and *rrnS*) and one large non-coding region [= a plausible control region (CR); 977 bp], as typically found in metazoa (Fig. 1 and Table 2). The components are compactly juxtaposed. There are even some overlapping nucleotides between *trnC *and *trnW *(1 bp), *nad3 *and *trnA *(3 bp), and *trnE *and *trnF *(1 bp) (Table 2). The order and orientation of the gene arrangement pattern is identical to that of a horseshoe crab, *Limulus polyphemus*, except for the position of the *trnQ *gene (Fig. 2).

**Table 1:** Comparison of whole genome sizes, A+T contents and GenBank accession numbers of the mitochondrial genomes examined in this study

Classification	Whole genome		Control region		GenBank Accession Numbers
		
	Size(bp)	A+T(%)	Size(bp), N^a^	A+T(%)	
**Subphylum Chelicerata**					
**Class Pycnogonida**					
*Achelia bituberculata*	15,474	77.0	977, 1	79.0	AY457170
*Nymphon gracile*	14,681	77.3	191, 1	85.3	NC_008572
**Class Arachnida**					
**Order Araneae**					
*Heptathela langzhouensis*	14,215	72.2	340, 1	80.6	NC_005924
*Habronattus oregonensis*	14,381	74.3	717, 1	77.1	NC_005942
*Ornithoctonus huwena*	13,874	69.8	396, 1	68.2	NC_005925
Order Scorpiones					
*Mesobuthus gibbosus*	15,983	67.0	1,434, 1	?	NC_006515
*Centruroides limpidus*	14,519	64.5	545, 1	60.9	NC_006896
Order Acari					
*Ixodes hexagonus*	14,539	72.7	359, 1	71.9	NC_002010
*Haemaphysalis flava*	14,686	76.9	310, 1	66.5	NC_005292
*Rhipicephalus sanguineus*	14,710	78.0	608, 2	67.0	NC_002074
*Amblyomma triguttatum*	14,740	78.4	614, 2	76.3	NC_005963
*Carios capensis*	14,418	73.5	342, 1	71.3	NC_005291
*Ornithodoros moubata*	14,398	72.3	342, 1	71.6	NC_004357
*Varroa destructor*	16,477	80.0	2,174, 1	79.7	NC_004454
**Class Merostomata**					
**Order Xiphosura**					
*Limulus polyphemus*	14,985	67.6	348, 1	81.3	NC_003067
**Subphylum Myriapoda**					
**Class Chilopoda**					
*Lithobius forficatus*	15,695	67.9	1,540, 1	77.0	NC_002629
*Scutigera coleoptrata*	14,868	63.7	479, 1	70.8	NC_005870
**Class Diplopoda**					
*Narceus annularus*	15,133	67.8	476, 1	64.7	NC_003343
*Thyropygus *sp.	14,922	69.4	479, 1	77.9	NC_003344
**Subphylum Crustacea**					
**Class Branchiopoda**					
*Artemia franciscana*	15,770	64.5	1,770, 1	68.0	NC_001620
*Daphnia pulex*	15,333	62.3	689, 1	67.0	NC_000844
**Class Malacostraca**					
*Penaeus monodon*	15,984	70.6	991, 1	81.5	NC_002184
*Pagurus longicarpus*	15,630	71.3	534, 1	70.0	NC_003058
**Subphylum Hexapoda**					
**Class Insecta**					
*Anopheles gambiae*	15,363	77.6	518, 1	94.2	NC_002084
*Locusta migratoria*	15,772	75.3	979, 1	86.0	NC_001712
*Triatoma dimidiata*	17,019	69.5	2,474, 2	67.1	NC_002609
*Drosophila melanogaster*	19,517	82.2	4,601, 1	95.5	NC_001709
*Tricholepidion gertschi*	15,267	68.6	397, 1	76.8	NC_005437
**Class Elipura**					
**Order Collembola**					
*Tetrodontophora*	15,455	72.7	955, 1	75.7	NC_002735
*Gomphiocephalus*	15,075	74.1	304, 1	86.5	NC_005438
**Phylum Ornychophora**					
**Family Peripatidae**					
*Epiperipatus biolleyi*	14,411	74.1	540, 1	82.4	NC_009082
**Phylum Mollusca**					
**Order Neoloricata**					
*Katharina tunicata*	15,532	69.4	424, 1	75.3	NC_001636
**Phylum Annelida**					
**Order Haplotaxida**					
*Lumbricus terrestris*	14,998	61.6	384, 1	64.3	NC_001673
**Order Phyllodocida**					
*Platynereis dumerilli*	15,619	64.1	1091, 1	71.9	NC_000931

^a ^The number of control regions within each mitochondrial genome

?: It is impossible to estimate the A+T content owing to the large number of unidentified sequences 'N'.

**Table 2:** The mitochondrial genome profile of a sea spider, *Achelia bituberculata*

Features	Positionon		Strand	Size (bp)	Codon		Intergenic nucleotides^a^
					
	From	To			Start	Stop	
*rrnL*	1	1210	-	1210	0		
*trnV*	1211	1277	-	58	0		
*rrnS*	1278	2053	-	776	0		
*trnQ*	2054	2121	-	68	0		
Control region	2122	3098	+	977	0		
*trnI*	3099	3162	+	64	0		
*trnM*	3173	3228	+	56	10		
*nad2*	3239	4220	+	982	ATG	T*	11
*trnW*	4221	4278	+	57	0		
*trnC*	4278	4339	+	61	-1		
*trnY*	4347	4403	+	57	7		
*cox1*	4406	5930	+	1525	GAT	T*	2
*cox2*	5931	6600	+	670	ATG	T*	0
*trnK*	6601	6668	+	68	0		
*trnD*	6669	6737	+	69	0		
*atp8*	6739	6874	+	136	ATA	T*	1
*atp6*	6876	7545	+	670	ATT	T*	1
*cox3*	7546	8334	+	789	ATG	TAA	0
*trnG*	8340	8409	+	70	5		
*nad3*	8410	8751	+	312	ATA	TAG	0
*trnA*	8749	8802	+	54	-3		
*trnR*	8803	8868	+	66	0		
*trnN*	8870	8936	+	67	1		
*trnS2*	8937	8995	+	59	0		
*trnE*	9001	9064	+	64	5		
*trnF*	9064	9128	-	64	-1		
*nad5*	9130	10833	-	1704	ATT	T*	1
*trnH*	10839	10909	-	71	5		
*nad4*	10914	12275	-	1362	ATG	TAA	4
*nad4L*	12281	12574	-	294	ATG	TA*	5
*trnT*	12580	12632	+	53	5		
*trnP*	12633	12709	-	77	0		
*nad6*	12712	13202	+	491	ATG	TA*	2
*cob*	13203	14325	+	1123	ATG	T*	0
*trnS1*	14326	14388	+	63	0		
*nad1*	14407	15339	+	933	ATA	TAG	18
*trnL1*	15340	15404	+	65	0		
*trnL2*	15410	15474	+	65	6		

^a ^indicates gap nucleotides (positive value) or overlapped nucleotides (negative value) between two adjacent genes.

* Incomplete termination codon, which is probably extended by post-transcriptional adenylation.

### Comparison of gene arrangements

As shown in Fig. 2, the mitochondrial gene arrangement of *A. bituberculata *was compared with that of *L. polyphemus*, which is considered a putative ground pattern for arthropods [38], and with those of two sea spiders, *N. gracile *and *E. spinosa*. The identical gene arrangement pattern shown in *A. bituberculata *and *L. polyphemus *except for the *trnQ *gene is an interesting feature because the other sea spider, *N. gracile*, has an extensive inversion (from *trnI *to *cox2*) and ten tRNA gene translocations (Fig. 2). The partial genome of *E. spinosa *has the same gene arrangement pattern as those of *L. polyphemus *and *A. bituberculata*.

**Figure 3:**
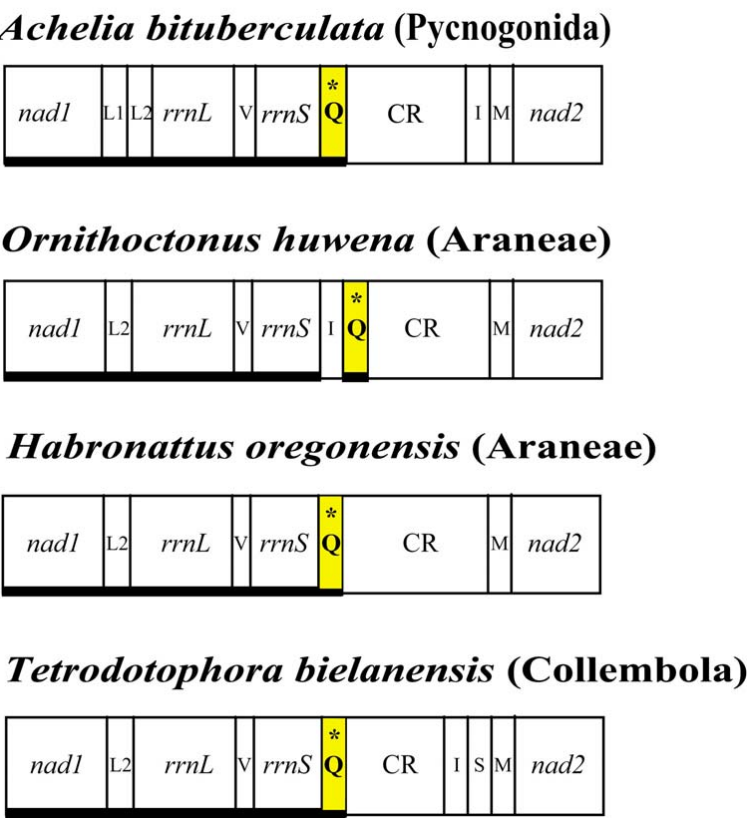
**Translocation of *trnQ *between the 12S rRNA (*rrnS*) and control region (CR) observed in a sea spider, *Achelia bituberculata*, two terrestrial spiders, *Ornithictinus huwena *and *Habronattus oregonensis*, and a collembolan, *Tetrodontophora bielanensis***. Compared to the typical arthropod *trnI-trnQ-M *arrangement between *nad2 *and the CR, the *trnQ *gene in *A. bituberculata *is found between *rrnS *and the CR. Such *trnQ *translocation appears in the two terrestrial spiders, *O. huwena *and *H. oregonensis*, and in a collembolan, *T. bielanensis*. Refer to the Fig. 1 legend for abbreviations of components. The Q position is highlighted with an asterisk (*) and a yellow box. Bold underlines indicate the reversed orientation of gene transcription.

The *trnQ *gene in *A. bituberculata *is located between *rrnS *and the control region (CR). It is found between *trnI*and *trnM *in most arthropods [38]. Comparisons among the *trnQ *positions in arthropods (Fig. 3) show that the *rrnS*-*trnQ-*CR arrangement in pycnogonids is also found in the jumping spider *Habronattus oregonensis *(Araneae, Salticidae) [47] and in a collembolan, *Tetrodontophora bielanensis *[48]. A similar pattern (*rrnS*-*trnI*-*trnQ*-CR) is also observed in the Chinese earth tiger spider *Ornithoctonus huwena *(Araneae, Theraphosidae) [47]. However, collembolans and spiders are not closely related, and another terrestrial spider (*Heptathela*) and another collembolan (*Gomphiocephalus hodgsoni*) have the *Limulus*-like arthropod ground pattern (*trnI-trnQ-trnM*). Thus, the *rrnS*-*trnQ*-CR arrangement found in a pycnogonid (*A. bituberculata*), a collembolan (*T. bielanensis*) and two terrestrial spiders (*H. oregonensis *and *O. huwena*) is likely to be homoplastic. Further intensive comparative studies are needed to examine whether the *rrnS*-*trnQ*-CR pattern is indeed a homoplastic feature or an important phylogenetic marker.

Furthermore, *A. bituberculata *and *N. gracile *have two different anticodon-possessing *trnL *genes, marked *L1 *and *L2*, which have the same orientations and positions as those in most other chelicerates and myriapods. Directly abutted tandem arrays of two *trnL*s between *rrnL *and *nad1 *(*rrnL-L1-L2-nad1*) are also observed in other metazoan phyla [49,50]. In Pancrustacea (= Hexapoda and Crustacea), *L1 *is generally located between *rrnL *and *nad1*, and *L2 *between *cox1 *and *cox2 *[50]. The *rrnL-L1-nad1 *and *cox1-L2-cox2 *arrangement patterns are considered to be critical evidence for a monophyletic origin of pancrustaceans other than the chelicerates and myriapods in arthropods. Therefore, the finding of the *rrnL-L1-L2-nad1 *arrangement in pycnogonids rejects the view that pycnogonids have crustacean affinities [5-9], generally regarded as an old-fashioned hypothesis today.

### Base composition and codon usage pattern

As shown in Table 1, the overall A+T content in the mitochondrial genome of *A. bituberculata *is 77.0% (+ strand: A = 38%; C = 12%; G = 11%; T = 39%). This A+T content is much higher than those in *L. polyphemus *(67.6%) and the scorpions (64.5 and 67.0%), but is similar to those in Acari and Araneae (69.8–80.0%), including *N. gracile *(76.6%).

Metazoan mitochondrial genomes usually present a clear strand bias in nucleotide composition [15,44]. Table 3 shows the AT- and CG-skews of each of the 13 protein-coding and 2 ribosomal RNA genes and those of the whole genome (total) in *A. bituberculata *mitochondria. The results show no marked strand-bias in nucleotide composition. The AT-skew for all genes is negative whether they are on the (+) or the (-) strand. The CG-skew is positive for four genes and negative for five on the (+) strand. The CG-skew for all six genes on the (-) strand is negative. This is totally different from the clear strand-bias observed in *N. gracile*.

**Table 3:** Nucleotide composition and skews of *Achelia bituberculata *mitochondrial protein-coding and ribosomal RNA genes

Gene	Proportion of nucleotides				AT%	AT skew	CG skew
	A	C	G	T			
*atp8 *(+)	0.388	0.075	0.068	0.469	85.7	-0.095	0.048
*atp6 *(+)	0.342	0.102	0.099	0.456	79.9	-0.143	0.015
*cox1 *(+)	0.287	0.139	0.165	0.408	69.6	-0.174	-0.087
*cox2 *(+)	0.338	0.126	0.134	0.402	74.0	-0.087	-0.033
*cox3 *(+)	0.304	0.115	0.154	0.427	73.1	-0.168	-0.146
*cob *(+)	0.346	0.141	0.108	0.405	75.1	-0.079	0.136
*nad1 *(-)	0.289	0.093	0.149	0.468	75.8	**-0.236**	**-0.230**
*nad2 *(+)	0.321	0.092	0.115	0.472	79.3	-0.190	-0.108
*nad3 *(+)	0.292	0.096	0.146	0.465	75.7	-0.228	-0.205
*nad4 *(-)	0.333	0.100	0.114	0.453	78.6	**-0.152**	**-0.061**
*nad4L *(-)	0.337	0.082	0.150	0.432	76.9	**-0.124**	**-0.294**
*nad5 *(-)	0.347	0.114	0.118	0.421	76.8	**-0.095**	**-0.015**
*nad6 *(+)	0.372	0.102	0.085	0.441	81.3	-0.085	0.087
*rrnL *(-)	0.369	0.075	0.138	0.417	78.7	**-0.061**	**-0.295**
*rrnS *(-)	0.380	0.079	0.138	0.403	78.4	**-0.030**	**-0.274**
Total (+)	0.379	0.119	0.110	0.391	77.0	-0.015	0.038

AT skew = (A%-T%)/(A%+T%); CG skew = (C%-G%)/(C%+G%); Bold letters mark the values of the genes on the (-) strand.

Investigation of the anticodon and codon usage patterns in the protein-coding genes of the *A. bituberculata *mitochondrial genome indicates that the nucleotides in the 3rd codon position are primarily 'A' or 'T' rather than 'C' or 'G'. Codons composed of only 'A' and 'T' are predominantly used, which seems to reflect the high A+T bias composition of the entire genome. A strong preference for A+T-rich codons is found in *A. bituberculata *as in mitochondrial protein-coding genes in general (Table 4). For example, the most frequently-used codons are L(UUA) (367 times) and I(AUU) (365 times). Additional file 1 shows the usage of leucine and serine codons in chelicerates and pycnogonids and reveals some interesting features in the usage of L(UUR) and L(CUN). Totals of 415 and 414 L(UUR) codons are used in *A. bituberculata *and *N. gracile*, respectively, which are the highest usage patterns of these codons in the chelicerates observed to date. A total of 125 L(CUN) codons are used in *A. bituberculata *(73 in *N. gracile*), similar to the usage patterns in Araneae and Acari (105~214) but different from those in *L. polyphemus *(259) and scorpions (241~287). That is, the L(CUN) codon frequency in *A. bituberculata *is similar to that in Araneae and Acari.

**Table 4:** Codon usage pattern of 13 mitochondrial protein-coding genes of a sea spider, *Achelia bituberculata*

Amino acid	Codon	N*	Amino acid	Codon	N*	Amino acid	Codon	N*	Amino acid	Codon	N*
Phe	UUU	290	Ser	UCU	104	Tyr	UAU	159	Cys	UGU	23
(GAA)	UUC	39	(UGA)	UCC	13	(GUA)	UAC	28	(GCA)	UGC	7
Leu	UUA	367		UCA	101	Ter	UAA	2	Trp	UGA	81
(UAA)	UUG	48		UCG	7		UAG	2	(UCA)	UGG	10
Leu	CUU	57	Pro	CCU	62	His	CAU	61	Arg	CGU	15
(UAG)	CUC	4	(UGG)	CCC	11	(GUG)	CAC	6	(UCG)	CGC	1
	CUA	54		CCA	41	Gln	CAA	50		CGA	29
	CUG	10		CCG	5	(UUG)	CAG	8		CGG	4
Ile	AUU	365	Thr	ACU	87	Asn	AAU	176	Ser	AGU	39
(GAU)	AUC	33	(UGU)	ACC	8	(GUU)	AAC	40	(UCU)	AGC	9
Met	AUA	288		ACA	37	Lys	AAA	94		AGA	63
(CAU)	AUG	75		ACG	6	(UUU)	AAG	14		AGG	17
Val	GUU	84	Ala	GCU	64	Asp	GAU	56	Gly	GGU	65
(UAC)	GUC	9	(UGC)	GCC	12	(GUC)	GAC	10	(UCC)	GGC	9
	GUA	85		GCA	33	Glu	GAA	60		GGA	84
	GUG	23		GCG	3	(UUC)	GAG	14		GGG	34

* The number of codons used in 13 mitochondrial protein-coding genes

### Protein-coding genes

The aggregate length of all the 13 protein-coding genes is 11,112 bp [A+T 8,446 bp (76%), G+C 2,666 bp (24%)]. There was no overlap between these genes. They account for 76% of the entire mitochondrial genome length. In terms of the lengths of individual protein-coding genes, *A. bituberculata *has relatively short *atp8 *(45 aa) and *nad3 *(104 aa) compared to other chelicerates (including *N. gracile*) and myriapods [See Additional file 2]. In contrast, the *nad4 *gene of *A. bituberculata *(454 aa) and *N. gracile *(460 aa) and *nad6 *of *A. bituberculata *(163 aa) are larger than those in the other species. While *nad6 *is dramatically reduced in *N. gracile *(125 aa), that in *A. bituberculata *is similar in size (163 aa) to the other chelicerates (141~158 aa).

Amino acid usages and A+T contents of mitochondrial protein-coding genes were compared with those of chelicerates [See Additional file 1]. Greater numbers of Asn (216 aa) and Met (363) are used than in the other chelicerates. The A+T content of the third codon positions of the 13 protein-coding genes is 85.99%, which is lower than in *N. gracile *(90.50%) and higher than in *L. polyphemus *(74.70%).

The start and stop codons of the 13 protein-coding genes of the *A. bituberculata *mitochondrial genome were identified by comparison with those of other known arthropods; particular attention was given to the amino acid sequence alignment in order to identify the most likely codon in ambiguous situations. As shown in Table 2, the start codons of 12 mitochondrial of the protein-coding genes (excluding *cox1*) are 'ATN', which is typical of most metazoan mitochondria [43]: 'ATG' is the start codon for *nad2*, *nad4L*, *nad6*, *cox2*, *cox3 *and *cob*; 'ATT' is the start codon found in *nad5 *and *atp6*; and 'ATA' is the start codon for *nad1*, *nad3*, *nad4 *and *atp8*. A less typical 'GAT' start codon appears in the remaining *cox1 *gene. On the other hand, only four of the protein-coding genes are terminated with a complete stop codon: 'TAA' in *nad4L *and *cox3 *and 'TAG' in *nad1 *and *nad3*. The remaining eight genes are terminated using truncated stop codons that are presumably completed by post-transcriptional polyadenylation [51]: 'TA' for *nad4 *and *nad6*, and 'T' for *nad2*, *nad5*, *cox1*, *cox2*, *cob*, *atp6 *and *atp8*.

### Transfer RNA genes

**Figure 4:**
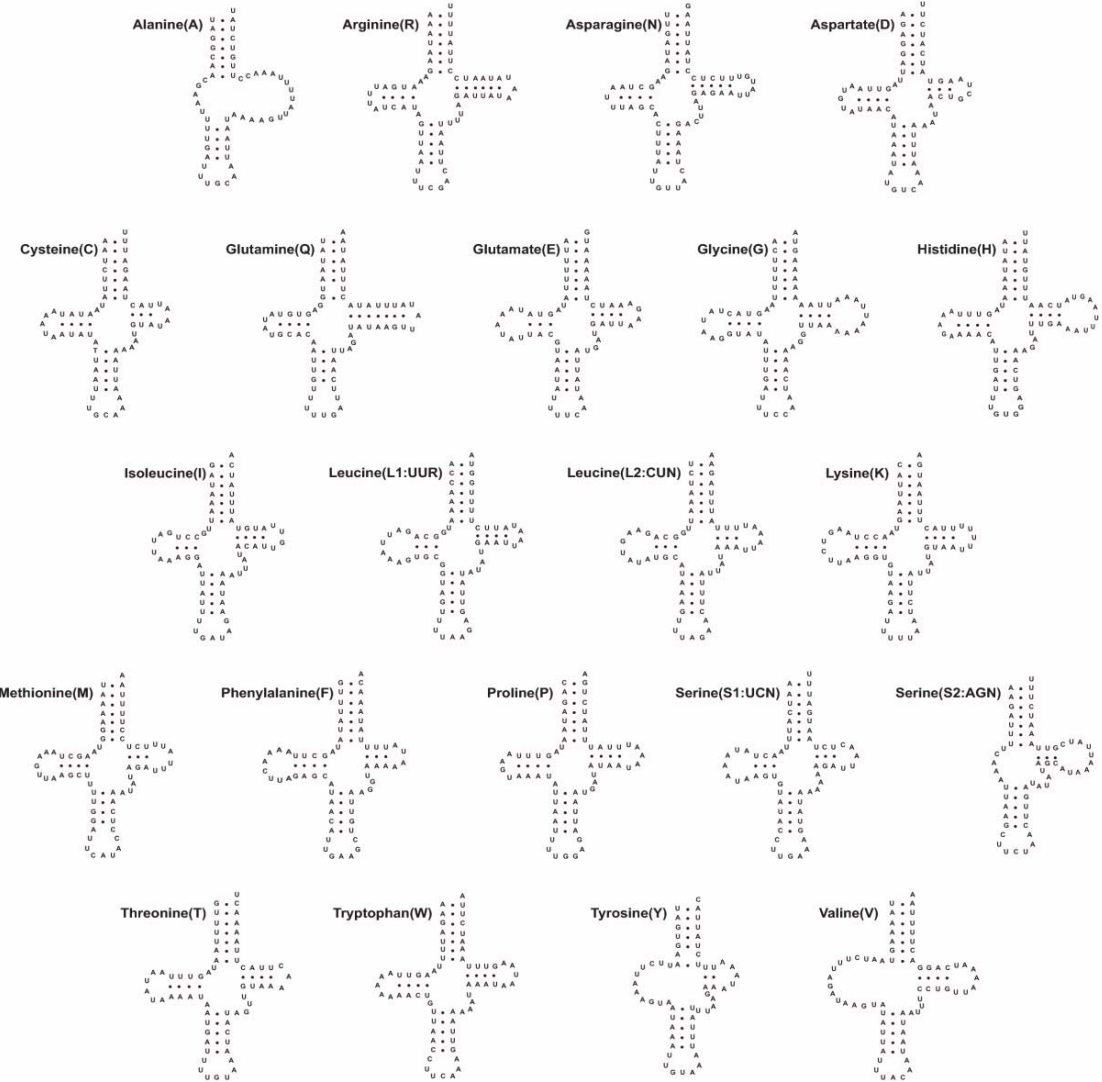
Putative secondary structures of the 22 tRNAs identified in the mitochondrial genome of the sea spider *Achelia bituberculata*.

All 22 of the tRNAs typically found in metazoan mitochondrial genomes were identified in *A. bituberculata*. All these genes are located among protein- and/or rRNA-coding genes (Fig. 1 and Table 2). Twenty-two putative secondary structures were predicted from the tRNA gene sequences (Fig. 4). Most of the tRNAs are capable of forming the typical clover-leaf structure, with the exception of tRNA^Ala ^(which lacks both the DHU and TψC arms), tRNA^Ser(AGN)^, tRNA^Tyr ^and tRNA^Val ^(which lacks only the DHU arm). The lengths of the TψC and DHU arms in the 18 tRNAs with a stable clover-leaf shape range from 4 to 12 bp, and the variable loops range in size from 4 to 5 bp. tRNA^Ser(AGN) ^with an unpaired DHU arm has been reported in the mitochondrial genomes of a number of metazoa including the sea spider *N. gracile *[44], the centipede *Scutigera coleoptrata *[52], the jumping spider *Habronattus oregonensis *[47], the nematodes *Caenorhabditis elegans *and *Ascaris suum *[53] and the annelid *Lumbricus terrestris *[39]. The DHU arm of tRNA^Ala ^in *N. gracile *does not form a stable stem; however, the other two tRNAs, tRNA^Tyr ^and tRNA^Val^, are folded into typical clover-leaf shapes [44].

Twenty of the 22 tRNAs have a completely matched 7-bp-long amino-acyl stem, and the remaining two, tRNA^Glu ^and tRNA^Tyr^, have a single mismatch within the stem. While 19 of the tRNAs have the typical 5-bp-long anticodon stem, the remaining three (tRNA^Ala^, tRNA^Glu^, and tRNA^Met^) have a single mismatch within this stem. It is not uncommon for some mitochondrial tRNAs to have stem mismatches, which are probably repaired by a post-transcriptional editing process [54].

The tRNA anticodon sequences in *A. bituberculata *(Table 4) are identical to those of the corresponding tRNAs of other metazoans with the exception of tRNA^lys ^and tRNA^Ser(AGN)^. tRNA^lys ^and tRNA^Ser(AGN) ^in both *A. bituberculata *and *N. gracile *possess 'UUU' and 'UCU' anticodons, respectively, instead of 'CUU' and 'GCU', which are more commonly found in invertebrates and some vertebrates [44,55].

### Ribosomal RNA genes

The *trnV *gene separates *rrnS *from *rrnL*, as in most arthropods. The sizes of *rrnS *and *rrnL *are approximately 1,210 bp and 776 bp, respectively [See Additional file 3]. These are similar to the centipedes *Lithobius forficatus *(1,188 bp and 763 bp) and *Scutigera coleoptrata *(1192 bp and 766 bp), but are slightly shorter than those of *L. polyphemus *(1,296 bp and 799 bp). Compared with that in arachnids, the length of *rrnL *in *A. bituberculata *is similar to those in four species of Acari (1,199 bp in *Amblyomma triguttatum*, 1,214 bp in *Ixodes holocyclus*, 1,212 bp in *Ornithodoros moubata *and 1,190 bp in *Rhipicephalus sanguinensis*) and larger than those in three terrestrial spiders (1,048 bp in *O. huwena*, 1,018 bp in *H. oregonensis *and 1,101 bp in *H. hangzhouensis*). On the other hand, *rrnS *in *A. bituberculata *is larger than those in four species of Acari (693 bp in *A. triguttatum*, 716 bp in *I. holocyclus*, 686 bp in *O. moubata*, 687 bp in *R. sanguineus*), and three terrestrial spiders (668 bp in *O. huwena*, 619 bp in *H. oregonensis*, 698 bp in *H. hangzhouensis*). The A+T contents of *rrnL *and *rrnS *are 78.7% (A, 505 nts; T, 447 nts) and 78.0% (A, 313 nts; T, 295 nts), respectively. These account for over 77% of the entire *A. bituberculata *mitochondrial genome. The *rrnL *gene of *A. bituberculata *has the highest similarities with those of *N. gracile *(59.5%) and *L. polyphemus *(57.3%), and the *rrnS *gene of *A. bituberculata *with that of *L. polyphemus *(50.1%).

### Control region

**Figure 5:**
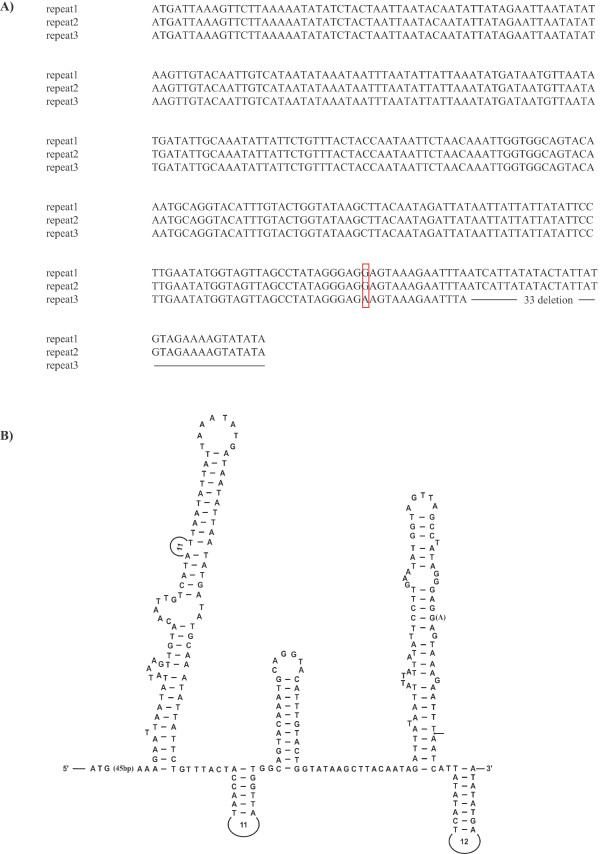
**Primary and secondary structures of the control region in the mitochondrial genome of the sea spider *Achelia bituberculata***. **(A) **The nucleotide sequence alignment of three repeat units observed in the control region of the mitochondrial genome of *A. bituberculata*. The lengths of the first and second repeat units are 315 bp. The 3'-end of the third repeat is truncated by 33 bp, resulting in a shorter repeat unit (282 bp). A red box indicates a single nucleotide difference observed in the third repeat unit. Dashes indicate truncated nucleotide sequences absent from the third repeat unit. **(B) **Putative secondary structure predicted from a large repeat unit (315 bp). An arrow marks the position truncated in the third repeat unit. An 'A' in parentheses indicates that a 'G' appears in the first and second repeat units, but an 'A' appears in the third repeat unit in position 3057 [GenBank: AY457170].

A large noncoding region [= a plausible control region (CR); 977 bp] is found between *trnQ *and *trnI*. It includes three tandem repeats, the first and second of which are completely identical in length (315 bp) and sequence (Fig. 5A). However, the third repeat (282 bp) is 33 bp shorter than the other two at the 3'-end, and one nucleotide is different in the 315-bp repeat (Fig. 5A). The repeats have unique stem and loop structures that may play key roles in controlling the replication and transcription of the mitochondrial genome (Fig. 5B). The A+T content of the CR is 79%, which is similar to those of most chelicerates (Table 1).

### The inclusion of Pycnogonida within Arachnida

**Figure 6:**
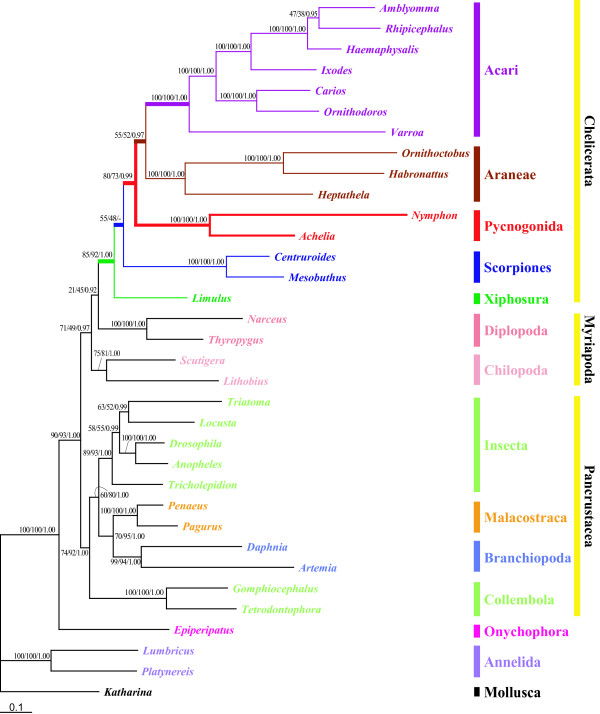
**Maximum likelihood tree inferred from amino acid residues deduced from 12 mitochondrial protein-coding genes of 30 arthropods and 4 non-arthropods**. The pycnogonids *Achelia bituberculata *and *Nymphon gracile *appear within the monophyletic Chelicerata with high node confidence values. Scorpiones are placed as the basal arachnid group. The inclusion of Pycnogonida within Arachnida is shown in this tree. The monophylies of Pancrustacea (Crustacea plus Hexapoda) and Myriochelata or Paradoxopoda (Myriapoda and Chelicerata) are strongly supported. The numbers above/below the branches indicate the node confidence values: BP_ML _(bootstrap proportion in the maximum likelihood analysis), BP_BI _(bootstrap proportion in Bayesian inference) and BPP (Bayesian posterior probability) in order. The tree was obtained from a final alignment 2232 aa sites in length (refer to Materials and Methods for more detailed information). The log likelihood value of the best tree is -72575.641555. For more detail information about the taxon, refer to Table 1.

As shown in Fig. 6, phylogenetic analyses based on the amino acid sequences of 12 protein-coding genes for 30 arthropods and 4 outgroups (1 onychophoran, 2 annelids and 1 mollusk) as shown in Table 1 indicate that Pycnogonida, including *A. bituberculata *and *N. gracile*, are clustered together (BP_ML _= 100, BP_BI _= 100, BPP = 1.0), and located within the monophyletic Chelicerata (BP_ML _= 85, BP_BI _= 92, BPP = 1.0). *L. polyphemus *appears to be a sister of all the other chelicerates (= Arachnida). Although a monophyletic Arachnida (Scorpiones, Araneae and Acari) including Pycnogonida is supported by node confidence values that are very low (BP_ML _= 55, BP_BI _= 48), Scorpiones are placed as the most basal arachnids and Pycnogonida, Araneae and Acari form a monophyletic group (BP_ML _= 80, BP_BI _= 73, BPP = 0.99); Pycnogonida is identified as a sister group of the clade of Araneae and Acari within Arachnida.

When the onychophoran *Epiperipatus biolleyi *is employed as a single outgroup, the resultant tree [See Additional file 4] is the same as Fig. 6 in the position of pycnogonids within Arachnida, although arthropod monophyly is not supported. The two pycnogonids examined, *A. bituberculata *and *N. gracile*, are clustered together (BP_ML _= 100, BP_BI _= 100, BPP = 1.0) within the monophyletic Chelicerata (BP_ML _= 92, BP_BI _= 92, BPP = 1.0). Pycnogonida again appears as a sister group of the clade of Araneae and Acari (BP_ML _= 72, BP_BI _= 73, BPP = 0.99).

Two previous studies based on the mitochondrial genome sequences of *E. spinosa *[15] and *N. gracile *[44] indicated that the Pycnogonida-Acari affinity might have resulted from biased features such as the strand-biased nucleotide contents and long-branch attraction. Interestingly, the *A. bituberculata *mitochondrial genome shows no marked strand-bias nucleotide composition (Table 3). Since most of the other chelicerates and *N. gracile *have strand-biased nucleotide contents [44]as well as extremely long branches on the phylogenetic tree (Fig. 6), the possibility remains that the appearance of Pycnogonida within Arachnida may result from artifacts of analysis due to the strand-biased and long-branching patterns.

To test whether the inclusion of Pycnogonida within Arthropoda results from long-branch attraction artifacts, we removed 9 long-branch taxa – all 7 Acari members and 2 terrestrial spiders (*H. oregonensis *and *O. huwena*) – from the dataset for the tree in Additional file 4. 1, and then conducted a new phylogenetic analysis with 21 ingroup and 1 outgroup taxa [See Additional file 5A]. We then removed *N. gracile *as well because of its long branching pattern, and reconstructed a new phylogenetic tree with only 20 ingroup and 1 outgroup taxa [See Additional file 5B]. In addition, we removed the 7 pancrutacean taxa and 1 onychophoran from the data sets including the 22 and 21 taxa, and then reconstructed additional phylogenetic trees with only 14 and 13 taxa, respectively [See Additional file 5C and 5D]. In the subsequent ML analyses, while Onychophora was used as an outgroup for the first and second reduced datasets including 22 and 21 taxa, the 4 remaining pancrustaceans were used as outgroups for the analyses with only 14 and 13 taxa. Although the long-branch taxa were removed, the ML trees inferred from the four different reduced alignment data sets showed that the pycnogonids are consistently clustered with the terrestrial spider *H. langzhouensis *[See additional file 5A, C, D], except for the ML tree with the 21 taxa in which *A. bituberculata *appears as a sister group of euchelicerates [See Additional file 5B].

The present findings conflict with the two major hypotheses that Pycnogonida is a sister group of the monophyletic Euchelicerata (= Arachnida + Xiphosura) or that it is a sister group of the monophyletic Cormogonida (= Euchelicerata + Myriapoda + Pancrustacea). Nevertheless, the inclusion of Pycnogonida within Arachnida is compatible with some previous analyses based on combinations of 253 morphological characteristics and molecular data (complete 18S rDNA and the D3 region of 28S rDNA) [14], and the mitochondrial protein-coding genes [15,44], which indicated that Pycnogonida appears within Arachnida. More recently, Mallatt and Giribet [31] on the basis of nearly-complete 18S and 28S rDNA sequences, and Giribet et al. [32] on the basis of parsimony analyses of combined data for 7 nuclear genes, 2 mitochondrial genes and 375 morphological characters, both showed that Pycnogonida is united with Chelicerata with very weak support and Cormogonida is strongly rejected. The present result is consistent with theirs in that Cormogonida is rejected and Pycnogonida has an affinity with Chelicerata. However, they did not suggest that Pycnogonida may possibly be included within Arachnida; many morphological taxonomists may not agree with this point of view. Thus, it is likely that the inclusion of Pycnogonida within Arachnida, deduced only from mitochondrial genome data [15,44], will remain an open question.

In addition, Scorpiones are often considered to be "one of the most primitive arachnids" because they possess a number of plesiomorphic character states, even though their phylogenetic position remains controversial. Recent publications based on mitochondrial protein-coding genes [56,57] show that Scorpiones is a sister group of all other arachnids (Acari and Araneae). The present result (Fig. 6) is consistent with previous reports [56,57] showing that Scorpiones is a sister group of the clade of Pycnogonida plus Araneae plus Acari.

The tree in Fig. 6 shows the paraphyly of Myriapoda [58]; the placement of Collembola as a sister of all other pancrustacean taxa [41]; the monophyly of Myriapoda and Chelicerata (= Myriochelata or Paradoxopoda) [15,16,40,59]; and the monophyly of Pancrustacea [15,16,19,50]. Complete mitochondrial genome sequences need to be determined from another important sister group (Tardigrada) of the arthropods in order to elucidate the deep arthropod phylogeny, including the phylogenetic position of pycnogonids.

## Conclusion

This study presents the complete sequence of the mitochondrial genome from a pycnogonid, *A. bituberculata *(Family Ammotheidae). This complete sequence and a previously-reported partial sequence of *Endeis spinosa *show the typical gene arrangement patterns of arthropods (*Limulus*-like), but they differ from that of *N. gracile*. Thus, it is most likely that the peculiar arrangements found in *N. gracile *are only present in the family Nymphonidae or even in the genus *Nymphon*. Phylogenetic trees based on amino acid sequences deduced from mitochondrial protein-coding genes showed that Pycnogonida may be authentic arachnids (= aquatic arachnids) within Chelicerata, as indicated by the name 'sea spider', and suggest that the Cormogonida theory, which considers the pycnogonids a sister of all other arthropods, should be rejected. However, since node confidence values supporting the Pycnogonida inclusion within Arachnida are slightly low and the possibility of long-branch attraction artifact still remains, further more intensive studies seem necessary to resolve the problem.

## Methods

### Specimen collection and DNA extraction

The pycnogonid *A. bituberculata *was collected from the intertidal zone of the East Sea (Pohang) in Korea. This specimen was immediately fixed in 70% ethanol and brought to our laboratory where it was preserved in absolute ethanol and stored at -20°C. The specimen was washed with distilled water, and total cellular DNA was extracted from a single individual using the DNeasy Tissue Kit (QIAGEN Co., Germany).

### PCR amplification and cloning

The entire mitochondrial genome was amplified by two polymerase chain reactions (PCR) using the following previously-reported primers: 16SB 5'-CCG GTT GAA CTC AGA TCA-3' [60] and HCO2198 5'-TAA ACT TCA GGG TGA CCA AAA AAT-3' [61] for 11 kb amplification, and 16SA 5'-CGC CTG TTT ATC AAA AAC AT-3' [62] and LCO1490 5'-GGT CAA CAA ATC ATA AAG ATA TTG G-3' [61] for 5 kb amplification. PCR amplification was performed using the Expand High Fidelity PCR Kit (Roche Co., Germany). The PCR protocol was as follows: 1 cycle at 94°C for 2 min; 10 cycles at 94°C for 15 s, 50°C for 30 sc, 68°C for 5~13 min; 20 cycles at 94°C for 15 s, 50°C for 30 s, 68°C for 5~13 min; 1 cycle at 68°C for 7 min. The PCR reactants were loaded on a 0.7% agarose gel and stained with ethidium bromide to visualize the bands using an ultraviolet transilluminator. PCR products of approximately 5 kb (*rrnL*-*cox1*) and 11 kb (*cox1*-*rrnL*) covering the entire mitochondrial genome were observed. The two PCR products were purified using the PCR Purification Kit (QIAGEN Co., Germany) and were ligated with the pGEM T-easy vector (Promega Co., USA). The ligation mixtures were transformed into *Escherichia coli *host strain DH5-α. Correct recombinant clones were selected by the blue/white colony selection method using X-gal and IPTG. Plasmid DNA was extracted using the AtmanBio plasmid purification kit (Takara Co., Japan), which was used for all subsequent sequencing reactions.

### Sequencing and sequence analysis

Both strands of the purified plasmid DNA were sequenced using a primer-walking strategy. The sequencing reactions were performed using the dideoxy-nucleotide termination method with the big-dye terminator system and an ABI3700 model automatic sequencer. The complete pycnogonid mitochondrial genome [GenBank: AY457170] was annotated using several stand-alone programs including Clustal X [63] and GeneJockey II v. 1.6 (Biosoft Inc., UK) and the BLAST web-based program. Thirteen protein-coding genes and two rRNA genes were identified by their sequence similarities to previously published genes. Potential secondary structures of 18 of the 22 tRNAs were identified using tRNAscan-SE v. 1.1 [64], and those of the other 4 by eye (*trnA*, *trnS2*, *trnY *and *trnV*).

### Phylogenetic analysis

For the present phylogenetic analyses we employed 30 arthropod ingroup taxa and 4 outgroup taxa, as listed in Table 1. The amino acid sequences of the 13 protein-coding genes were used. The 13 multiple alignment subsets of these sequences were created using a Clustal X multiple alignment program [63] under the default option. Only well-aligned, conserved alignment sites were extracted from each alignment subset using the Gblock program [65] with the default option. The *atp8 *gene was excluded from the following analyses because it is very variable in sequence and too short in length, so no region was selected by the Gblock program. The extracted conserved blocks were subsequently concatenated into a unified, single large alignment set with the Gblock program. Phylogenetic analyses inferred from nucleotide sequences are not presented because the resultant trees are unresolved in deep branches in spite of removing 3^rd^-codon positions. It is likely that amino acid sequences are considered more appropriate for resolving deep branching patterns in this study.

The refined alignment (2232 aa positions in length) was subjected to two different tree-making algorithms: the maximum likelihood (ML) and Bayesian inference (BI) methods. Rather than using hierarchical likelihood ratio tests to select the best fitting model for the evolution of sequences, and to calculate the related parameter values (I and Γ), ProtTest ver. 1.3 [66] was used under Akaike Information Criterion (AIC) because it has several important advantages [67]. Among the 36 models implemented in this program, the best-fitting model selected was mtREV [68] with among-site substitution-rate heterogeneity described by a gamma distribution (Γ = 0.86) and a fraction of sites constrained to be invariable (I = 0.19). The ML analysis was carried out using PHYML v2.4.4 [69]. The bootstrap proportions (BP_ML_; 100 replicates) of the ML tree were obtained by the fast-ML method using PHYML. BI analysis was carried out using the MrBayes v3.0b4 program [70] with the following options: 1,000,000 generations, 8 chains (2 hot and 6 cold) and a burn-in step of the first 50,000. Node confidence values of the BI tree were presented with Bayesian posterior probabilities (BPP). We applied bootstrap re-sampling procedures to the Bayesian approach since it is known that posterior probabilities place excessive confidence on a given phylogenetic hypothesis. Bayesian bootstrap proportions (BP_BI_) were calculated as follows: 100 bootstrapped datasets were generated with the SEQBOOT program under the PHYLIP package Version 3.6b [71], and then each dataset was analyzed by MrBayes v3.0b4 [70], with four independent Markov chains run for 500,000 Metropolis-coupled MCMC generations, with tree sampling every 100 generations and a burn-in of 10,000 trees. Finally, the 100 Bayesian majority rule consensus trees were used to construct the BBP consensus tree with the CONSENSUS program using the PHYLIP package Version 3.6b [71].

## Abbreviations

*atp6 *and *atp8*, genes for the ATPase subunits 6 and 8; *cox1*-*cox3*, genes for cytochrome C oxidase subunits I-III; *cob*, a gene for apocytochrome b; *nad1*-*nad6 *and *nad4L*, genes for NADH dehydrogenase subunits 1–6 and 4L; *rrnS *and *rrnL*, genes for 12S and 16S rRNAs. *trnX*, where X is replaced by single-letter amino acid abbreviations of the corresponding amino acids. *L1 *and *L2*, genes for tRNA^Leu(UUR) ^(anticodon TAA) and tRNA^Leu(CUN) ^(anticodon TAG), respectively; *S1 *and *S2*, genes for the tRNA^Ser(UCN) ^(anticodon TGA) and tRNA^Ser(AGN) ^(anticodon GCT), respectively. ML, the maximum likelihood method; BI, Bayesian inference; BPP, Bayesian posterior probabilities; BP_ML _and BP_BI_, bootstrap proportions in maximum likelihood and Bayesian inference analyses, respectively.

## Competing interests

The author(s) declares that there are no competing interests.

## Authors' contributions

SJP and UWH made substantial contributions to the conception and design of the study, acquisition of the data, and analysis and interpretation of the data. SJP wrote the early draft of this manuscript, and UWH revised and rewrote all parts of the manuscript. YSL helped to analyze the data. All authors have read and approved the final version of the manuscript. UWH gave final approval of the version to be published.

## Supplementary Material

Additional file 1 Amino acid usage and AT content of mitochondrial protein-coding genes from various arthropods. Click here for file

Additional file 2 Length comparison of the 13 mitochondrial protein-coding genes of a sea spider, *Achelia bituberculata*, with those of some representative chelicerates and myriapods. Click here for file

Additional file 3 Length comparison of *rrnL *and *rrnS *between the chelicerates and myriapods used in this study. Click here for file

Additional file 4 **Maximum likelihood tree inferred from amino acid residues deduced from 12 mitochondrial protein-coding genes of 30 arthropods and 1 onychophoran**. From Fig. 6, other non-arthropods (1 mollusk and two annelids) are removed, and *Epiperipatus biolleyi *(Onychophora) was used as an outgroup. The log likelihood value of the best tree is -68554.411179. For more detail information, refer to Table 1 and Fig. 6. Click here for file

Additional file 5 **Four maximum likelihood trees inferred from the four different types of reduced alignment sets of amino acid sequences deduced from the 12 mitochondrial protein-coding genes**. To reduce long-branch artifacts, most of the taxa with long branches such as acaris, two terrestrial spiders, *Nymphon gracile*, and so on. (A) 22 taxa – 21 arthropods and 1 onychophoran outgroup without 7 acaris and 2 terrestrial spiders (log likelihood value = -56915.290653); (B) 21 taxa – 20 arthropods and 1 onychophoran outgroup without *Nymphon gracile*, 7 acaris and 2 terrestrial spiders (log likelihood value = -56797.452176); (C) 14 arthropod taxa – removal of 7 pancrustaceans and 1 onychophoran from the (A) dataset (log likelihood value = -41668.552731); (D) 13 arthropod taxa – additional removal of *N. gracile *from the (C) dataset (log likelihood value = -40520.848760). The numbers above the branches are the node confidence values obtained with 100 bootstrap replicates. The PhyML program was used to reconstruct the ML trees. In (A) and (B), an onychophoran, *Epiperipatus biolleyi*, is used as an outgroup, and in (C) and (D), 4 pancrustaceans are used. For details of species names and classification, refer to Table 1. Click here for file

## References

[B1] Munilla T, Melic A, De Haro JJ, Mendez M, Ribera IZ (1999). Evolución y filogenia de los picnogónidos. Evolución y Filogenia de Arthropoda.

[B2] Weygoldt P, Paulus HF (1979). Untersuchungen zur Morphologie, Taxonomie und Phylogenie der Chelicerata. Z Zool Syst Evolut Forsch.

[B3] Brusca RC, Brusca GJ, Brusca RC, Brusca GJ (2003). Chelicerata. Invertebrates (2nd).

[B4] Dunlop JA, Arango CP (2005). Pycnogonid affinities: a review. J Zool Syst Evol Res.

[B5] Savigny IC (1816). Théorie des Organes de la Bouche des Crustacés et des Insectes, I, II.

[B6] Krøyer H (1840). Om Pycnogonidernes Forvandlinger. Naturhistorik Tidsskrift, Kjobenhavn.

[B7] Eights J (1835). Description of a new animal belonging to the Arachnides of Lateille; Discovered in the sea along the shores of the New South Shetland Islands. Bost J Nat Hist.

[B8] Costa OG (1836). Aracnidi. Famiglia II. Picnogonidi. Fauna del Regno di Napoli.

[B9] Krøyer H (1845). Birdrag til Kundskab om Pycnogoniderne eller Sospindlerne. Naturhistorik Tidsskrift, Kjobenhavn.

[B10] Thompson DAW, Harmer SF, Shipley AE (1901). Pycnogonida. The Cambridge Natural History.

[B11] Stømer L (1944). On the relationship and phylogeny of fossil and recent Arachnomorpha. Skrifer utgitt av Det Norske Videnskaps-Akademi i Oslo I Mat-Naturv Klasse.

[B12] King PE (1973). Pycnogonids.

[B13] Lankester ER (1905). The structure and classification of the Arachnoidea. Subclass I: Pantopoda. Qt J Microscop Sci N S.

[B14] Giribet G, Edgecombe GD, Wheeler WC, Babbitt C (2002). Phylogeny and systematic position of opiliones: A combined analysis of chelicerate relationshps using morphological and moleucular data. Cladistics.

[B15] Hassanin A (2006). Phylogeny of Arthropoda inferred from mitochondrial sequences: Strategies for limiting the misleading effects of multiple changes in pattern and rates of substitution. Molecular phylogenetics and evolution.

[B16] Mallatt JM, Garey JR, Shultz JW (2004). Ecdysozoan phylogeny and Bayesian inference: first use of nearly complete 28S and 18S rRNA gene sequences to classify the arthropods and their kin. Molecular phylogenetics and evolution.

[B17] Regier JC, Shultz JW, Kambic RE (2005). Pancrustacean phylogeny: hexapods are terrestrial crustaceans and maxillopods are not monophyletic. Proceedings.

[B18] Zrzavý J, Hypsa V, Vlásková M, Fortey RA, Thomas RH (1998). Arthropod phylogeny: taxonomic congruence, total evidence and conditional combination approaches to morphological and molecular data sets. Arthropod Relationships.

[B19] Giribet G, Edgecombe GD, Wheeler WC (2001). Arthropod phylogeny based on eight molecular loci and morphology. Nature.

[B20] Maxmen A, Bowne WE, Martindale MQ, Giribet G (2005). Neuroanatomy of sea spiders implies an appendicular origin of the protocerebral segment. Nature.

[B21] Ax P (1984). Das Phylogenetische System.

[B22] Weygoldt P (1986). Arthropod interrelationship: the phylogenetic-systematic approach. Z Zool Syst Evolut Forsch.

[B23] Wheeler WC, Cartwright P, Hayashi CY (1993). Arthropod phylogeny: a combined approach. Cladistics.

[B24] Wheeler WC, Hayashi CY (1998). The phylogeny of the extant chelicerate orders. Cladistics.

[B25] Walossek D, Müller KJ, Fortey RA, Thomas RH (1998). Arthropod Relationships. Cambrian 'Orsten'-type arthropods and the phylogeny of Crustacea.

[B26] Shultz JW, Regier JC (2000). Phylogenetic analysis of two nuclear protein-encoding genes in arthropods supports a crustacean-hexapod clade. Proc R Soc Lond B.

[B27] Regier JC, Shultz JW (2001). Elongation factor-2: a useful gene for arthropod phylogenetics. Molecular phylogenetics and evolution.

[B28] Walossek D, Dunlop JA (2002). A larval sea spider (Arthropoda: Pycnogonida) from the Upper Cambrian 'Orsten' of Sweden, and the phylogenetic position of pycnogonids. Palaeontology.

[B29] Jager M, Murienne J, Clabaut C, Deutsch J, Le Guyader H, Manuël M (2006). Homology of arthropod anterior appendages revealed by Hox gene expression in a sea spider. Nature.

[B30] Manuël M, Jager M, Murienne J, Clabaut C, Guyader HL (2006). Hox genes in sea spiders (Pycnogonida) and the homology of arthropod head segments. Dev Genes Evol.

[B31] Mallatt J, Giribet G (2006). Further use of nearly complete 28S and 18S rRNA genes to classify Ecdysozoa: 37 more arthropods and a kinorhynch. Molecular phylogenetics and evolution.

[B32] Giribet G, Richter S, Edgecombe GD, Wheeler WC, Koenemann S, Jenner RA (2005). The position of crustaceans within Arthropoda - Evidence from nine molecular loci and morphology. Crustacea and Arthropod relationships.

[B33] Edgecombe G, Wilson GDF, Colgan DJ, Gray MR, Cassis G (2000). Arthopod cladistics: combined analysis of histone H3 and U2 snRNA sequences and morphology. Cladistics.

[B34] Giribet G, Ribera C (2000). A review of arthropod phylogeny: new data based on ribosomal DNA sequences and direct character optimization. Cladistics.

[B35] Budd G (2002). A palaeontological solution to the arthropod head problem. Nature.

[B36] Scholtz G, Edgecombe GD (2005). Heads, Hox and the phylogenetic position of trilobites. Crustac Issues.

[B37] Boore JL, Brown WM (2000). Mitochondrial genomes of Galathealinum, Helobdella, and Platynereis: sequence and gene arrangement comparisons indicate that Pogonophora is not a phylum and Annelida and Arthropoda are not sister taxa. Mol Biol Evol.

[B38] Boore JL (1999). Animal mitochondrial genomes. Nucleic Acids Res.

[B39] Boore JL, Brown WM (1998). Big trees from little genomes: mitochondrial gene order as a phylogenetic tool. Curr Opin Genet Dev.

[B40] Hwang UW, Friedrich M, Tautz D, Park CJ, Kim W (2001). Mitochondrial protein phylogeny joins myridpods with chelicerates. Nature.

[B41] Nardi F, Spinsanti G, Boore JL, Carapelli A, Dallai R, Frati F (2003). Hexapod origins: monophyletic or paraphyletic?. Science.

[B42] Hwang UW, Kim W (1999). General properties and phylogenetic utilities of nuclear ribosomal DNA and mitochondrial DNA commonly used in molecular systematics. Korean J Parasitol.

[B43] Wolstenholme DR (1992). Genetic novelties in mitochondrial genomes of multicellular animals. Curr Opin Genet Dev.

[B44] Podsiadlowski L, Braband A (2006). The complete mitochondrial genome of the sea spider Nymphon gracile (Arthropoda: Pycnogonida). BMC Genomics.

[B45] Arango CP (2002). Morphological phylogenetics of the sea spiders (Arthropoda: Pycnogonida). Org Div Evol.

[B46] Arango CP (2003). Molecular approach to the phylogenetics of sea spiders (arthropoda: pycnogonida) using partial sequences of nuclear ribosomal DNA. Molecular phylogenetics and evolution.

[B47] Masta SE, Boore JL (2004). The complete mitochondrial genome sequence of the spider Habronattus oregonensis reveals rearranged and extremely truncated tRNAs. Mol Biol Evol.

[B48] Nardi F, Carapell A, Fanciulli PP, Dallai R, Frati F (2001). The complete mitchondrial DNA sequence of the basal hexapod Testrodontophora bielanensis: evidence for heteroplasmy and tRNA translocations. Mol Biol Evol.

[B49] Boore JL, Collins TM, Stanton D, Daehler LL, Brown WM (1995). Deducing the pattern of arthropod phylogeny from mitochondrial DNA rearrangements. Nature.

[B50] Boore JL, Lavrov DV, Brown WM (1998). Gene translocation links insects and crustaceans. Nature.

[B51] Ojala D, Montoya J, Attardi G (1981). tRNA punctuation model of RNA processing in human mitochondria. Nature.

[B52] Negrisolo E, Minelli A, Valle G (2004). Extensive gene order rearrangement in the mitochondrial genome of the centipede Scutigera coleoptrata. J Mol Evol.

[B53] Okimoto R, Macfarlane JL, Clary DO, Wolstenholme DR (1992). The mitochondrial genomes of two nematodes, Caenorhabditis elegans and Ascaris suum. Genetics.

[B54] Lavrov DV, Brown WM, Boore JL (2000). A novel type of RNA editing occurs in the mitochondrial tRNAs of the centipede Lithobius forficatus. Proc Natl Acad Sci U S A.

[B55] Yamauchi MM, Miya MU, Nishida M (2002). Complete mitochondrial DNA sequence of the Japanese spiny lobster, Panulirus japonicus (Crustacea: Decapoda). Genetics.

[B56] Jones M, Gantenbein B, Fet V, Blaxter M (2007). The effect of model choice on the phylogenetic position of scorpions inferred from mitochondrial genes. Molecular phylogenetics and evolution.

[B57] Choi EH, Park SJ, Jang KH, Hwang UW (2007). Complete mitochondrial genome of a Chinese scorpion Mesobuthus martensii (Chelicerata, Scorpiones, Buthidae). DNA sequence.

[B58] Negrisolo E, Minelli A, Valle G (2004). The mitochondrial genome of the house centipede Scutigera and the monophyly versus paraphyly of myriapods. Mol Biol Evol.

[B59] Friedrich M, Tautz D (1995). Ribosomal DNA phylogeny of the major extant arthropod classes and the evolution of myriapods. Nature.

[B60] Kambhampati S, Smith PT (1995). PCR primers for amplification of four insect mitochondrial gene fragments. Insect Mol Biol.

[B61] Folmer O, Black M, Hoeh R, Lutz RA, Vrijenhoek R (1994). DNA primers for amplification of mitochondrial cytchrome C oxidase subunit I from diverse metazoan invertebrates. Mol Mar Biol Biotechnol.

[B62] Simon C, Frati F, Beckenach A, Crespi B, Liu H, Flook P (1994). Evolution, weighting and phylogenetic utility of mitochondrial gene sequences and a compilation of conserved polymerase chain reaction primers. Ann Entomol Soc Amer.

[B63] Thompson JD, Gibson TJ, Plewniak F, Jeanmougin F, Higgins DG (1997). The Clustal X windows interface: flexible strategies for multiple sequence alignment aided by quality analysis tools. Nucleic Acids Res.

[B64] Lowe TM, Eddy SR (1997). tRNA-SE: a program for improved detection of transfer RNA genes in genomic sequence. Nucleic Acids Res.

[B65] Castresana J (2000). Selection of conserved blocks from multiple alignments for their use in phylogenetic analysis. Mol Biol Evol.

[B66] Abascal F, Zardoya R, Posada D (2005). ProtTest: selection of best-fit models of protein evolution. Bioinformatics.

[B67] Posada D, Buckley T (2004). Model selection and model averaging in phylogenetics: advantages of Akaike information criterion and Bayesian approaches over likelihood ratio tests. Syst Biol.

[B68] Adachi J, Hasegawa M (1996). Model of amino acid substitution in proteins encoded by mitochondrial DNA. J Mol Evol.

[B69] Guindon S, Gascuel O (2003). A simple, fast, and accurate algorithm to estimate large phylogenies by maximum likelihood. Syst Biol.

[B70] Huelsenbeck JP, Ronquist F (2001). MRBAYES: Bayesian inference of phylogenetic trees. Bioinformatics.

[B71] Felsenstein J (2004). PHYLIP (Phylogeny Inferenece Package) version 3.6b. Department of Genome Sciences, University of Washington Seattle.

