# Supplementary material for: The complete mitochondrial genome of the sea spider Achelia bituberculata (Pycnogonida, Ammotheidae): arthropod ground pattern of gene arrangement
Source: BMC Genomics. 2007 Oct 1;8:343. doi: 10.1186/1471-2164-8-343 (PMC2194727; doi:10.1186/1471-2164-8-343)
Supplement: Additional file 1 — Amino acid usage and AT content of mitochondrial protein-coding genes from various arthropods. [file 1471-2164-8-343-S1.doc]

**Additional file 1.** Amino acid usage and AT content of mitochondrial protein-coding genes from various arthropods

| **Taxon** | Species | **Ala** | **Arg** | **Asn** | **Asp** | **Cys** | **Gln** | **Glu** | **Gly** | **His** | **Ile** | **Leu** | **Lys** | **Met** | **Phe** | **Pro** | **Ser** | **Thr** | **Trp** | **Tyr** | **Val** | **AT%** | **AT%** |
| --- | --- | --- | --- | --- | --- | --- | --- | --- | --- | --- | --- | --- | --- | --- | --- | --- | --- | --- | --- | --- | --- | --- | --- |
|  |  | GCN | CGN | AAY | GAY | TGY | CAR | GAR | GGN | CAY | ATY | TTR | AAR | ATR | TTY | CCN | TCN | CAN | TGR | TAY | GTN | PCG | PCG |
|  |  |  |  |  |  |  |  |  |  |  |  | CTN |  |  |  |  | AGN |  |  |  |  | total | 3^rd^ pos. |
| Acari | *Ornithodoros mou.* | **123** | 51 | 163 | 57 | **33** | 66 | 87 | 207 | 71 | 386 | 511 | **115** | **288** | 368 | 131 | 318 | 186 | 99 | 130 | 209 | 71.30% | 78.50% |
|  | *Rhipicephalus san.* | **117** | **44** | **206** | 58 | **34** | 56 | 79 | **163** | 65 | **462** | 487 | **136** | **318** | 394 | 120 | 355 | 142 | **80** | 129 | **139** | 77.90% | **89.50%** |
|  | *Varroa destructor* | **100** | 48 | **210** | 54 | **29** | **39** | 90 | **160** | 65 | 412 | 507 | **118** | **350** | 367 | **113** | 317 | **123** | **82** | **189** | **163** | 79.20% | **91.70%** |
|  | *Amblyomma trig.* | **103** | **41** | **200** | 52 | **31** | 51 | 83 | **161** | 63 | **494** | 469 | **141** | **311** | 436 | **106** | 352 | 154 | **80** | 130 | **130** | 78.30% | 88.40% |
|  | *Haemaphysalis fl.* | **109** | **42** | **190** | 52 | **33** | **48** | 81 | **163** | 69 | **488** | 476 | **137** | **328** | 389 | **113** | 362 | 140 | **82** | 135 | **147** | 76.60% | 85.50% |
|  | *Carios capensis* | **119** | 50 | 158 | 63 | **32** | 59 | 81 | 207 | 69 | 414 | 536 | **120** | **293** | 364 | 139 | 339 | 150 | 96 | 131 | 172 | 72.50% | 79.90% |
|  | *Ixodes hexagonus* | **115** | 48 | 161 | 64 | **32** | **49** | 79 | 181 | 67 | **436** | 495 | **118** | **296** | 385 | 137 | 385 | 140 | 95 | 121 | 182 | 71.10% | 75.70% |
| Araneae | *Habronattus oreg.* | 138 | 52 | 152 | 67 | **24** | **48** | 89 | 206 | 67 | 362 | 494 | 84 | **338** | 317 | 116 | 386 | **134** | 95 | **158** | 234 | 73.80% | 86.10% |
|  | *Ornithoctonus huw.* | 147 | 53 | **109** | **74** | **23** | **48** | 96 | 213 | 73 | 358 | 469 | 97 | **273** | 355 | 134 | 402 | 139 | 99 | 127 | 260 | 69.70% | 78.40% |
|  | *Heptathela han.* | 135 | 53 | 136 | 54 | **29** | 64 | 90 | 218 | 73 | 369 | 561 | 100 | **274** | 344 | 140 | 375 | 151 | 100 | 114 | 192 | 71.40% | 82.10% |
| Scorpiones | *Mesobuthus* | **107** | 61 | **96** | 68 | **34** | 59 | **178** | 231 | 64 | **168** | 544 | **195** | **145** | 313 | **98** | 322 | **84** | 102 | 98 | 251 | 67.00% | 70.40% |
|  | *Centruroides lim.* | 190 | 63 | **93** | 65 | 43 | **46** | 86 | 253 | 77 | 250 | 604 | 79 | 184 | 355 | 145 | 376 | 176 | 103 | 120 | 298 | 62.90% | 68.10% |
| Pycnogonida | *Nymphon gracile* | **125** | 53 | 160 | 69 | **35** | **46** | 80 | 188 | 71 | 362 | 494 | **123** | **286** | 420 | 136 | 370 | 158 | **82** | 139 | 218 | 76.60% | **90.50%** |
|  | *Achelia bituber.* | **112** | 49 | **216** | 66 | **30** | 58 | 74 | 192 | 67 | 398 | 540 | **108** | **363** | 329 | 119 | 353 | 138 | 91 | **187** | 201 | 76.08% | 85.99% |
| Xiphosura | *Limulus polyph* | 176 | 62 | 147 | 59 | 49 | 67 | 86 | 237 | 78 | 346 | 565 | 83 | 212 | 330 | 152 | 387 | 178 | 110 | 121 | 221 | 66.30% | 74.70% |

Bold numbers indicate strong differences (+/-25%) from *Limulus polyphemus* (underlined).
