# Supplementary material for: The complete mitochondrial genome of the sea spider Achelia bituberculata (Pycnogonida, Ammotheidae): arthropod ground pattern of gene arrangement
Source: BMC Genomics. 2007 Oct 1;8:343. doi: 10.1186/1471-2164-8-343 (PMC2194727; doi:10.1186/1471-2164-8-343)
Supplement: Additional file 2 — Length comparison of the 13 mitochondrial protein-coding genes of a sea spider, Achelia bituberculata, with those of some representative chelicerates and myriapods. [file 1471-2164-8-343-S2.doc]

**Additional file 2.** Length comparison of the 13 mitochondrial protein-coding genes of a sea spider, *Achelia bituberculata*, with those of some representative chelicerates and myriapods

| PCG* |  | The number of amino acid residues | | | | | |
| --- | --- | --- | --- | --- | --- | --- | --- |
|  | *Achelia* | *Nymphon* | *Limulus* | *Ixodes* | *Heptathela* | *Lithobius* | *Thyropygus* |
| *atp6* | 223 | 204 | 222 | 220 | 220 | 222 | 225 |
| *atp8* | **45** | 52 | 51 | 51 | 49 | 51 | 51 |
| *cox1* | 508 | 512 | 511 | 512 | 510 | 511 | 510 |
| *cox2* | 223 | 228 | 227 | 225 | 222 | 227 | 227 |
| *cox3* | 263 | 262 | 262 | 261 | 257 | 261 | 261 |
| *cob* | 374 | 374 | 376 | 366 | 373 | 377 | 372 |
| *nad1* | 311 | 304 | 300 | 313 | 304 | 311 | 305 |
| *nad2* | 327 | 327 | 320 | 318 | 320 | 325 | 335 |
| *nad3* | **104** | 115 | 117 | 111 | 109 | 117 | 115 |
| *nad4* | **454** | **460** | 444 | 436 | 433 | 443 | 442 |
| *nad4L* | 98 | 93 | 93 | 91 | 93 | 93 | 91 |
| *nad5* | 568 | 558 | 569 | 554 | 545 | 569 | 570 |
| *nad6* | **163** | **125** | 153 | 141 | 143 | 158 | 153 |

For the species names and their classification, refer to Table 1.

*Protein-coding genes
