# Supplementary material for: The complete mitochondrial genome of the sea spider Achelia bituberculata (Pycnogonida, Ammotheidae): arthropod ground pattern of gene arrangement
Source: BMC Genomics. 2007 Oct 1;8:343. doi: 10.1186/1471-2164-8-343 (PMC2194727; doi:10.1186/1471-2164-8-343)
Supplement: Additional file 3 — Length comparison of rrnL and rrnS between the chelicerates and myriapods used in this study. [file 1471-2164-8-343-S3.doc]

**Additional file 3.** Length comparison of *rrnL* and *rrnS* between the chelicerates and myriapods used in this study

| Classification | *rrnL*(bp) | *rrnS*(bp) |
| --- | --- | --- |
| **Subphylum Chelicerata** |  |  |
| **Class Pycnogonida** |  |  |
| ***Achelia bituberculata*** | **1210** | **776** |
| *Nymphon gracile* | 1196 | 767 |
| **Class Arachnida** |  |  |
| Order Araneae |  |  |
| *Heptathela langzhouensis* | 1101 | 698 |
| *Habronattus oregonensis* | 1018 | 691 |
| *Ornithoctonus huwena* | 1048 | 666 |
| Order Scorpiones |  |  |
| *Mesobuthus gibbosus* | 1147 | 762 |
| *Centruroides limpidus* | 1132 | 727 |
| Order Acari |  |  |
| *Ixodes hexagonus* | 1287 | 705 |
| *Haemaphysalis flava* | 1196 | 699 |
| *Rhipicephalus sanguineus* | 1190 | 687 |
| *Amblyomma triguttatum* | 1199 | 693 |
| *Carios capensis* | 1225 | 695 |
| *Ornithodoros moubata* | 1212 | 686 |
| *Varroa destructor* | 1149 | 726 |
| **Class Merostomata** |  |  |
| Order Xiphosura |  |  |
| *Limulus polyphemus* | 1296 | 799 |
| **Subphylum Myriapoda** |  |  |
| **Class Chilopoda** |  |  |
| *Lithobius forficatus* | 1188 | 763 |
| *Scutigera coleoptrata* | 1192 | 766 |
| **Class Diplopoda** |  |  |
| *Narceus annularus* | 1291 | 784 |
